# Will hypolimnetic waters become anoxic in all deep tropical lakes?

**DOI:** 10.1038/srep45320

**Published:** 2017-03-28

**Authors:** Takehiko Fukushima, Bunkei Matsushita, Luki Subehi, Fajar Setiawan, Hendro Wibowo

**Affiliations:** 1 Faculty of Life and Environmental Sciences, University of Tsukuba, 1-1-1 Tennodai, Tsukuba, Ibaraki 305-8572, Japan; 2 Research Center for Limnology, LIPI, Kompleks LIPI-Cibinong Km 46, Cibinong 16911, Indonesia

## Abstract

To elucidate trends of hypolimnetic oxygen concentrations, vertical distributions of dissolved oxygen were measured in eight deep tropical bodies of water (one natural lake with two basins, five natural lakes, and one reservoir) in Indonesia. A comparison of those concentrations with previously reported data revealed that shoaling of hypolimnetic oxygen-deficient (around a few decimeters to a few meter per year) water had occurred in all of the lakes. Calculated areal hypolimnetic oxygen depletion rates were 0.046–5.9 g m^−2^ y^−1^. The oligomictic or meromictic characteristics of the bodies of water suppressed circulation and mixing in the hypolimnions and thus resulted in continuous shoaling of the uppermost oxygen-deficient layers. In some lakes, millions of fish sometimes died suddenly, probably owing to upward movement of oxygen-deficient water to near the surface during periods of strong winds. In the future, the rate of shoaling will be accelerated by human impacts in the basins and by climate warming, the influence of which has already been manifested by rising water temperatures in these lakes. Appropriate monitoring and discussions of future restoration challenges are urgently needed to prevent the hypolimnions of the lakes from becoming completely anoxic.

Depletion of dissolved oxygen in the hypolimnions of lakes during stratification and its deleterious effects on fish stocks have been observed and analyzed for more than 100 years, especially in European and North American lakes^1^. On the basis of their analysis of sediments from 365 lakes worldwide, Jenny *et al*.^2^ reported that lacustrine hypoxia started spreading before AD 1900, 70 years prior to the occurrence of hypoxia in coastal zones, mainly as a result of an increase of human activities and associated nutrient releases.

Dramatic decreases in areal hypolimnetic oxygen deficits have been observed in several lakes due to reductions of phosphorus loading (e.g., Lake Spokane^3^). However, there has been a recurrence of eutrophication problems and hypoxia in the central basin of Lake Erie since the mid-1990s after the extent of hypoxia was reduced in response to load reductions initiated in 1972^4^. Recently, dozens of studies have indicated that climate warming accelerates deep-water hypoxia in temperate and subtropical lakes^5^,^6^,^7^,^8^.

Much less attention has been paid to the water quality of tropical lakes compared to temperate and subtropical lakes. Hecky *et al*.^9^ have reported that low oxygen concentrations (<1 mg l^−1^) were observed in the water of Lake Victoria at depths as shallow as 40 m in 1990–1991; in 1961 the shallowest occurrence of hypoxia was at depths >50 m. Recently, Cohen *et al*.^10^ reported the fish habitable area (>4 mg l^−1^) shrank rapidly (90 m in 1956, 80 m in 1993, 70 m in 2002, and 62 m in 2012) in Lake Tanganyika, Africa’s deepest and oldest lake in the tropics. In meromictic lakes, the behavior of methane has attracted scientists’ attention in lakes Hule and Rio Cuarto^11^ and Lake Kivu^12^. As a matter of course, it is crucial to know the long-term trend of lake oxygen concentrations in tropical lakes to facilitate sustainable use of lake water and conservation of endemic species.

A small number of papers have reported the oxygen concentrations of Indonesian lakes, which are located in the tropics. The German Limnological Sunda-Expedition of 1928–1929 reported the vertical distributions of lake water quality (e.g., water temperature, pH, and concentrations of dissolved oxygen and phosphorus) of 15 Indonesian lakes (eight lakes in Java, five lakes in Sumatra, and two lakes in Bali)^13^. In 1977, Lehmusluoto and Marbub^14^ measured selected physical and chemical parameters in the waters of three lakes on Bali and concluded that there had been very little change of the water quality of those three lakes during the intervening 48 years, although human activity had increased in catchment areas of the lakes. Luhmusluoto^15^ has subsequently reported the results of nation-wide limnological surveys of the physical and chemical characteristics of 24 lakes and 14 reservoirs in Indonesia during the period 1992–1993, but that report includes no discussion of the trends of lake characteristics^14^. Several elaborate surveys have subsequently been carried out in particular lakes^16^,^17^. Although trends of water quality, particularly oxygen concentrations, are therefore very intriguing, they have not been quantified for Indonesian lakes, which include some deep, oligomictic or meromictic lakes that are subject to stresses from human activities and climate warming.

In the present study, we measured the vertical distributions of water quality in 10 lakes and 2 reservoirs in Indonesia during the period 2011–2016. A comparison was then made with the above-mentioned published reports, particularly for dissolved oxygen and water temperature for six lakes (including one lake with two basins) and one reservoir (Table 1). Hereafter, we called the period during the year 1929 “A”, the period from 1975 to 2005 “B”, and the period from 2013 to 2016 “C”. Hypolimnetic areal oxygen depletion rates were then calculated to investigate their long-term changes. Finally, we speculate about the nature and magnitude of future challenges, particularly with respect to monitoring and rehabilitation.

## Results

**Figure 1:**
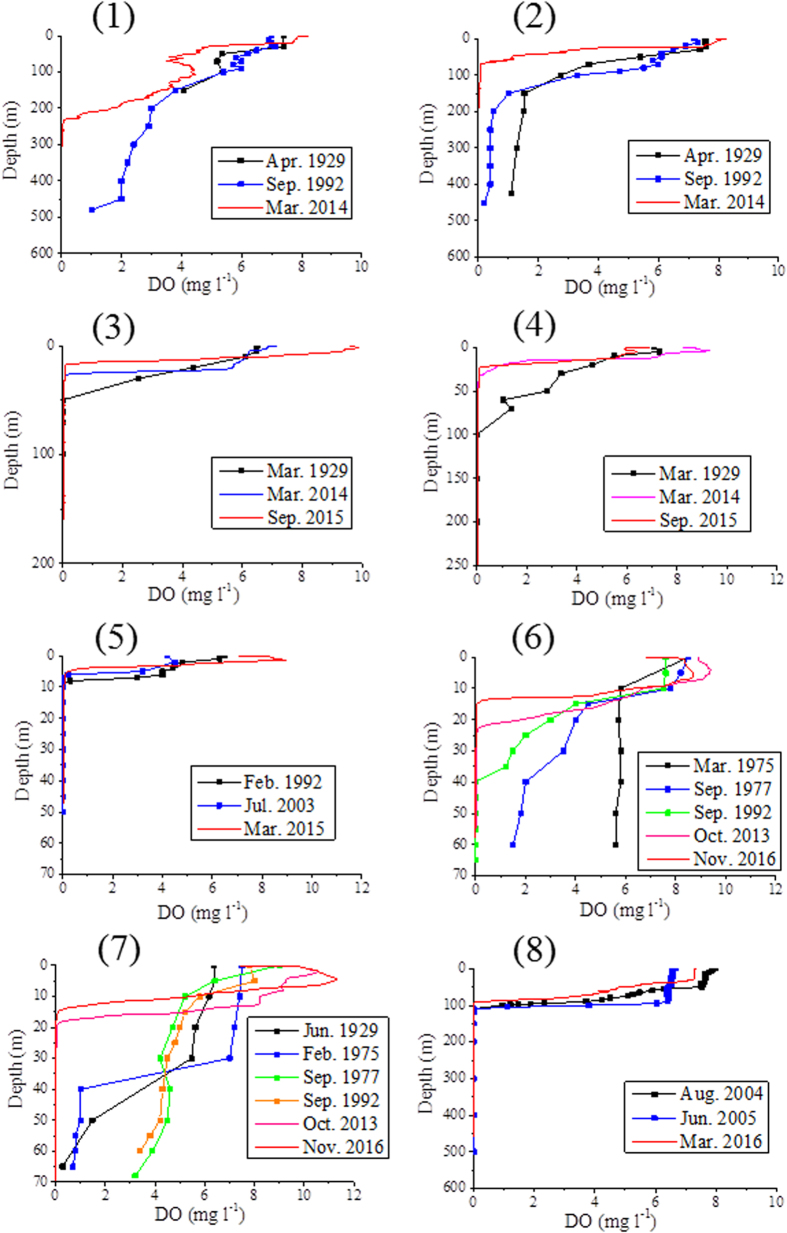
Changes in vertical profiles of dissolved oxygen concentrations. Solid squares indicate the measured values other than our sensor measurements. (1) Lake Toba North Basin, (2) Lake Toba South Basin, (3) Lake Maninjau, (4) Lake Singkarak, (5) Cirata Reservoir, (6) Lake Buyan, (7) Lake Batur, (8) Lake Matano.

Changes in the vertical profiles of dissolved oxygen are shown in Fig. 1. In all lakes, the hypolimnetic dissolved oxygen concentrations were lower in the present surveys (2011–2016) than in the past. Table 2 summarizes the shallowest depths of anoxic water (oxic-anoxic boundary). Anoxic layers were apparent in all of the lakes during the 2011–2016 survey, and the shallowest depths of anoxic water were shallower than in the past. Dissolved oxygen saturation percentages in these lakes are shown in Supplementary Fig. 1, indicating quite similar distributions to those of dissolved oxygen concentrations. Areal hypolimnetic oxygen depletion rates, which were calculated between the same seasons as explained below, showed clear increasing features with time (Table 3). Dissolve oxygen conditions in our surveyed lakes with no comparison data are shown in supplementary information. Anoxic waters were also observed in all of the lakes except Lake Limboto, which is a shallow lake with a maximum depth of 2.5 m^18^.

Water temperature increases of a few degrees were observed in the north and south basins of Lake Toba and in lakes Maninjau, Singkarak, Batur, and Matano (Supplementary Fig. 2). Differences in the patterns of temperature increase were apparent: a hypolimnetic temperature increase in Lake Toba, an epilimnetic temperature increase in Lake Matano, and a temperature increase throughout the water column in lakes Maninjau, Singkarak, and Batur. In contrast, decreases of water temperature were found during 2003–2015 in the Cirata Reservoir and during 1977–1992 in Lake Buyan.

## Discussion

**Figure 2:**
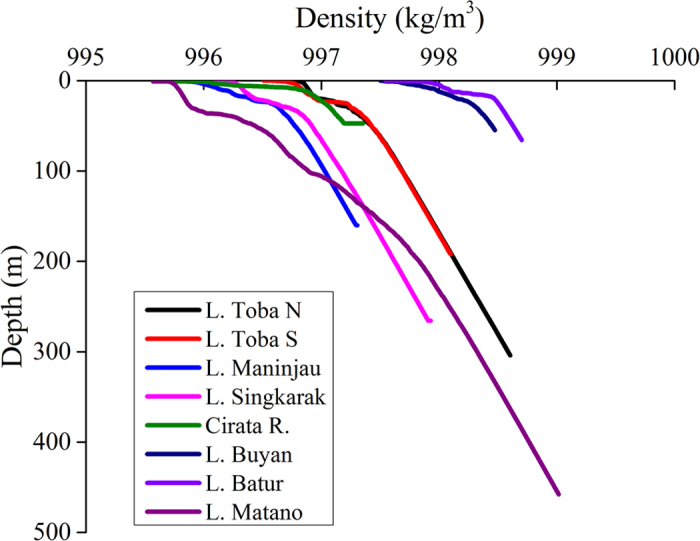
Vertical profiles of water density in the eight water bodies.

The vertical distributions of water density indicated stable stratification (Fig. 2). Supplementary Fig. 3 indicates clear density gradients (thermoclines) in these lakes, and a density difference of 1–2 kg m^−3^ was usual in the water columns of all the lakes. This order of density difference corresponds to a temperature change from 4 °C to 21 °C (2 kg m^−3^ difference), which is sometimes observed in summer in warm monomictic or dimictic deep lakes^19^. This is due to an exponential decrease in water density with temperature in higher water temperature range. Such differences probably depress mixing in the hypolimnion, and these lakes may then become oligomictic or meromictic^19^. Katsev *et al*.^20^ have indicated that the estimated timescale of water renewal in the monimolimnion (perennially isolated hypolimnion) of Lake Matano is several hundred years. In contrast, Lehmusluoto and Mahbub^14^ have suggested the need for further study of the circulation and stratification in lakes Buyan and Batur, which have relatively shallow depths and are located at high altitudes. In all the lakes except Lake Batur, electric conductivity showed increases with depth (Supplementary Fig. 4). This may be due to release of soluble ions from anoxic sediments (e.g., release of iron and manganese via redox reactions). However, its influence on density gradients was negligible compared with temperature and depth influences.

There is a possibility of circulation influence on the vertical profiles of dissolved oxygen in some of the lakes, where the circulation depends principally on seasonal meteorological patterns. Therefore, the comparison of dissolve oxygen profiles on a seasonal basis would be reasonable. After classifying the seasons in the respective lakes (see Method), Table 2 certainly indicates the shoaling of anoxic water even by the comparison on a seasonal basis. Anyway, seasonal changes in these vertical profiles should be monitored in the future to determine the circulation effect on the shoaling of anoxic water.

The shoaling rates of anoxic water in Indonesian deep lakes were around a few decimeters to a few meters per year (Table 2). These rates were similar to the observed ones in Lake Victoria^9^ and Lake Tanganyika^10^ as mentioned in the introduction. In Table 3, areal hypolimnetic oxygen depletion rates (AHOD) were calculated for only the differences between the same seasons. In Lakes Buyan and Batur, the differences within period C were calculated in spite of different seasons (see Method) because the sampling dates were quite close to each other. Increasing trends in AHOD were clearly seen in the calculated cases. Walker^21^ has reported AHOD values of 0.06–1.73 g m^−2^ d^−1^ in 24 Connecticut impoundments, 13 Canadian lakes, and 8 US lakes that ranged in trophic status from oligotrophic to hypereutrophic. They considered the periods of stratification in these lakes to be 200 d long; the yearly depletion rates were therefore 12–236 g m^−2^ y^−1^, which are much higher than the AHODs observed in Indonesian lakes (Table 3). Muller *et al*.^1^ have reported total areal hypolimnetic mineralization rates (including oxygen depletion and production of reduced materials) to be 0.47–1.31 g m^−2^ d^−1^ (corresponding to 94–262 g m^−2^ y^−1^) for French and Swiss eutrophic lakes; those rates also greatly exceed the values in Table 3 (0.046–5.9 g m^−2^ y^−1^). The French and Swiss lakes are located in the temperate zone, and overturning of the whole water column would therefore be expected to occur once or twice a year. The supply of oxygen-rich waters to the hypolimnion could result in higher rates of decomposition of organic substances and consequently higher AHODs. In deep Indonesian lakes, the slow diffusion of dissolved oxygen from the epilimnion and the slow diffusion of reduced materials from the sediments may account for their small AHODs compared with temperate lakes.

O’Reilly *et al*.^22^ reported that lake summer surface water temperature rose rapidly (global mean: 0.34 °C decade^−1^) between 1985 and 2009 using *in situ* and satellite-derived lake data. Although they showed that surface water warming rates were dependent on a combination of climate and local characteristics, rather than just lake location, the increased rates in tropical lakes were generally lower than temperate lakes. The Indonesian Climate Change Sectorial Roadmap^23^ has reported that an air temperature increase of about 0.5 °C occurred during the 20^th^ century, based on monthly surface air temperatures collected from a limited number of stations in Indonesia over a period of 100 years. A similar temperature increase has also been reported by Case *et al*.^24^. The increases of water temperature observed in this study are comparable in magnitude to those of air temperature, the indication being that the increases were influenced by global warming.

In contrast, the reason(s) for the observed decreases in water temperature are somewhat unclear. In the Cirata Reservoir, the water temperature and the amount of water inflow could affect the heat budget in the reservoir to a greater extent than in the cases of natural lakes, the result possibly being a decrease of water temperature. The decrease of temperature from 1977 to 1992 in Lake Buyan might be due to partial or complete mixing, which can happen unexpectedly at high altitudes during the relatively cooler period of the northeasterly monsoon^15^, because this lake is relatively shallow and is located at an altitude of 1217 m. In these lakes, a moderate supply of dissolved oxygen from inflow waters and surface exchange is a possibility that should be considered.

Jacobson^25^,^26^ has reported the sudden deaths of millions of fish in Lakes Maninjau and Toba. A sudden depletion of oxygen was considered to be the cause, together with unfavorable weather conditions and unsustainable practices by local aquaculturists. A check should therefore be made to determine whether or not hypolimnetic oxygen-deficient water has moved upward to the surface of these lakes. For example, the following conditions related to the thermocline and wind are assumed in Lake Maninjau: epilimnetic water temperature, 29 °C; hypolimnetic water temperature, 27 °C; depth of thermocline, 20 m; fetch (length), 16 km; density of air, 1.21 kg m^−3^; and wind friction coefficient, 1.2 × 10^–3^. The magnitude of the windward thermocline elevation and leeward thermocline drop can be calculated using the equations (3) to (7) based on theoretical considerations by assuming a rectangular lake with constant depth, two layers of different density with no eddy viscosity along the boundary, and a stationary state (i.e., enough elapsed time after the start of the wind stress)^27^. In the case of Lake Maninjau, a wind speed faster than 10.4 m s^−1^ can elevate hypolimnetic water to the surface at the windward edge of the lake. Because this wind speed is not especially unusual, the upwelling of oxygen-deficient water to the surface is possible in this lake. Although the situation in other lakes is not as critical as in Lake Maninjau, other lakes also face the risk of a similar input of oxygen-deficient water in the near future.

Eutrophication is probably another reason for the shoaling of anoxic water in Indonesian lakes. The changes in Secchi disk transparency (Supplementary Table 2) indicate the deterioration of light conditions in these lakes through eutrophication. Particularly, in Lakes Toba (oligotrophic in the periods A and B to between oligotrophy and mesotrophy in the period C), Maninjau (between oligotrophy and mesotrophy in the period A to mesotrophic in the periods B and C) and Batur (Mesotrophic in the period B to eutrophic in the period C), clear tendencies of eutrophication were observed. However, the relative contributions of water temperature increase and eutrophication on the shoaling of anoxic water were not identified and remain a future challenge.

Assuming that the rates of shoaling of oxygen-deficient water and vertical mixing will not change in the future, all of the deep lakes in Indonesia may face a crisis of anoxic conditions throughout almost the entire hypolimnetic water column. There is a possibility that the shoaling rate may be accelerated by human impacts in the watershed through increasing organic and nutrient loads and climate change. The most serious aspect of this problem is that this shoaling is an irreversible phenomenon.

To avoid the development of such critical conditions, more effort is needed to acquire an accurate understanding of the current situation, trends, and mechanisms; new strategies for restoration will also be needed. To reduce the inputs of organic matter and nutrients, wastewater management as well as prohibition of excessive aquaculture will be indispensable. Adaptive water management in the basin and downstream area will be essential.

## Methods

### Our survey

During the period 2011–2016, we visited 12 lakes (Maninjau, Singkarak, Rawa Pening, Gajah Munghur, Tondano, Limboto, Tempe, Buyan, Batur, Toba, Matano, and Towuti in the chronological order of sampling) and three reservoirs (Saguling, Jatiluhur, and Cirata). Six lakes (including one lake with two basins) and one reservoir were chosen for further analysis based on the measured data shown in Table 1. The vertical profiles of water quality parameters (water temperature, electric conductivity, dissolved oxygen, turbidity, and chlorophyll-*a*) were measured at intervals of 10 cm or 20 cm with a multi–water quality profiler (RINKO-profiler, JFE Advantech Co., Ltd, Tokyo, Japan). Salinity was calculated as a function of electric conductivity by assuming the ionic composition of seawater and then water density was calculated as a function of water temperature, salinity and depth. Because the calculated salinity was rather low (maximum 1.0 in Lake Batur), the influence of salinity on water density was negligible. Water samples were taken for measurements of, inter alia, suspended solids, chlorophyll-*a*, and pH; Secchi depths were measured with a 30-cm-diameter white disc. The analyses in this study made use of the vertical profiles of dissolved oxygen, water temperature and electric conductivity measured with the profiler and the water densities and dissolved oxygen saturation percentages calculated therefrom. The precision and resolution (in parentheses) of the sensors were ±0.3% full scale (FS) (0.01 m) for depth estimated with the pressure sensor, ±0.01 °C (0.001 °C) for water temperature measured with the thermistor, and ±2% FS (less than 0.004 mg l^−1^) for dissolved oxygen measured via phosphorescence.

Judging from the measured Secchi depths, we calculated the trophic state indices to be 33, 49, 39, 54, 46, 56, and 19 for lakes Toba, Maninjau, Singkarak, Cirata, Buyan, Batur, and Matano, respectively^28^. Based on the criteria proposed by Carlson and Simpson^29^, Lake Matano was classified as oligotrophic, Lakes Toba and Singkarak were intermediate between oligotrophy and mesotrophy, Lakes Maninjau and Buyan were mesotrophic, and Cirata Reservoir and Lake Batur were eutrophic.

### Previous reports

Previously reported vertical profiles of dissolved oxygen and water temperature were used in the analysis (supplementary Table 1). Ruttner^13^ and Crowe *et al*.^17^ reported numerical values, but other studies have presented figures showing vertical distributions. In the latter cases, we estimated numerical values from the vertical profiles. Concentrations of dissolved oxygen were determined by modified Winkler’s method^30^ in Ruttner^13^, APHA/AWWA/WPCF^31^ in Lehmusluoto & Mahbub^14^, electrometric (galvanic) method in Lehmusluoto^15^, multi-parameter water quality meter (AAQ1183, Alec Electronics) in Hayami *et al*.^16^, and Clark-type electrode to a depth of 100 m and linear sweep voltammetry at deeper depths in Croweet *et al*.^17^. For the details, see the original materials.

### Classification of seasons in Indonesian lakes

Aldrian and Susanto^32^ proposed three climate zones based on climatic rainfall variability using the double correlation method (DCM). Region X is located southern Indonesia from Sumatera to Timor Island, southern Kalimantan, Sulawesi and part of Irian Jaya. Region Y is located in northern Indonesia from northern Sumatora to northwestern Kalimantan. Region Z encompasses Maluku and northern Sulawesi. Then, Lakes Toba, Maninjau and Singkarak belong to Region Y. Lakes Buyan, Batur and Matano and Cirata Reservoirbelong to Region X. Seasonal patterns of meteorological conditions in the respective lakes are expressed as below, considering seasonal changes in air temperature (https://tdjamaluddin.wordpress.com/2011/04/)^33^.

Lakes Toba, Maninjau and Singkarak: wet from September to December (Season 1), dry in other months (Season 2), and unclear seasonal change in air temperature.

Lakes Buyan, Batur and Matano: dry-cool from June to October (Season 1), wet-hot in other months (Season 2).

Cirata Reservoir: dry from June to October (Season 1), wet in other months (Season 2), and unclear seasonal change in air temperature.

### Areal hypolimnetic oxygen depletion rates

Areal hypolimnetic oxygen depletion rates (*AHOD*) were then calculated using the following equations:


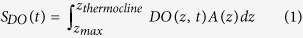



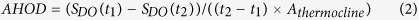


where *S*_*DO*_ (*t*) is the integral of dissolved oxygen beneath the thermocline at time *t, DO (z, t*) is the dissolved oxygen concentration at depth *z* and time *t, A(z*) is the area of the body of water at depth of *z, t*_1_ and *t*_2_ are two different times, and *A*_thermocline_ is the area of the body of water at the depth of the thermocline. Data on depth–area relationships were obtained from bathymetric maps of the respective bodies of water. Thermocline depths were determined roughly by using water temperature profiles (20 m for the north and south basins of Lake Toba; 20 m for Lake Maninjau and Lake Singkarak; 6 m for Cirata Reservoir; 10 m for Lake Buyan and Lake Batur; and 40 m Lake Matano).

### Calculation of thermocline elevation and drop

We consider^27^ the motion of water in a lake with a length of *l* and two layers of different density. It is blowing a wind with a speed of *W*. The surface elevation *η* in a stationary state is given by


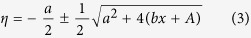


where 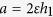, 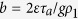 and *A* is a constant of integration. *ε* is relative difference of density as expressed by


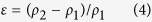


where *ρ*_1_ is the density of upper layer (above thermocline), *ρ*_2_ is that of lower layer (below thermocline), *h*_1_ is the averaged depth of upper layer, *τ*_a_ is the stress exerted on the water surface, and *g* is the gravitational acceleration. The constant *A* is to be determined from the conservation of mass such that


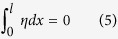


where *x* is the distance from the windward shore. *τ*_a_ is expressed by


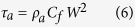


where *ρ*_*a*_ is the density of air and *C*_*f*_ is wind friction coefficient. Equations (3) and (5) were solved by iteration.

The positions of thermocline at windward shore is calculated as


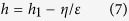


As wind blows faster, *h* gets closer to zero. When *h* is zero, *A* cannot be determined indicating the upward movement of hypolimnion to the surface.

## Additional Information

**How to cite this article**: Fukushima, T. *et al*. Will hypolimnetic waters become anoxic in all deep tropical lakes? *Sci. Rep.* **7**, 45320; doi: 10.1038/srep45320 (2017).

**Publisher's note:** Springer Nature remains neutral with regard to jurisdictional claims in published maps and institutional affiliations.

## Supplementary Material

Supplementary Information

## Figures and Tables

**Table 1:** Characteristics of analyzed lakes.

Lake name	Island	Area (km^2^)	Max depth (m)	elevation (m) 18	Origin^18^	Secchi depth (m)*
L. Toba North	Sumatra	538^13^	529^15^	905	Tecto-volcanic	6.5
L. Toba South	Sumatra	586^13^	433^15^	905	Tecto-volcanic	6.4
L. Maninjau	Sumatra	94^18^	165^18^	462	Tecto-volcanic	2.1
L. Singkara	Sumatra	108^18^	268^18^	362	Tectonic	4.4
Cirata R.	Jawa	62^18^	125^18^	206–220	Man-made	1.5
L. Buyan	Bali	3.7^18^	70^18^	1217	Caldera	2.6
L. Batur	Bali	15.9^18^	88^18^	1031	Caldera	1.3
L. Matano	Sulawesi	164^18^	590^18^	382	Tectonic	16.8

*Average of all measurements by this study.

**Table 2:** The shallowest depth of anoxic water (corresponding to Supplementary Table 1).

Lake name	A (1929)	B (1975–2005)			C (2013–2016)^*1^	
	No. 1	No. 1	No. 2	No. 3	No. 1	No. 2
L. Toba North	bottom (1929)	bottom (1992)			231 m (2014)	
L. Toba South	bottom (1929)	bottom (1992)			68 m (2014)	
L. Maninjau	>30 m (1929)				27.1 m (2014)	17.8 m (2015)
L. Singkarak	>70 m (1929)				32.8 m (2014)	23.5 m (2015)
Cirata R.		8 m (1992)	7 m (2003)		6.3 m (2015)	
L. Buyan		bottom (1975)	bottom (1975)	40 m (1992)	23.1 m (2013)	14.6 m (2016)
L. Batur	bottom (1929)	bottom (1975)	bottom (1977)	bottom (1992)	18.6 m (2013)	14.8 m (2016)
L. Matano		105 m (2005)	108 m (2006)		91.4 m (2016)	

Numbers indicate the order of measurements in the respective periods (A, B and C; see text). The underlined values indicate the sampling done in Season 1 and others in Season 2 (see Method).

*^1^Anoxic layer was defined as the water column of DO < 0.1 mgl^−1^ in our measurements.

**Table 3:** Areal hypolimnetic oxygen depletion rate (gO_2_ m^−2^ y^−1^).

Lake name	Depth of thermocline (m)	A (1929) to C (2013–2016)	B (1975) to C (2013–2016)	within C (2013–2016)
L. Toba North	20			
L. Toba South	20	0.046 (1929–2014)		
L. Maninjau	20	0.080 (1929–2014)		
L. Singkarak	20	0.20 (1929–2014)		
Cirata R.	6		0.47 (1992–2015)	
L. Buyan	10		1.1 (1977–2013), 0.52 (1992–2013)	5.8 (2013–2016)
L. Batur	10	0.64 (1929–2013)	1.7 (1977–2013), 3.0 (1992–2013)	4.7 (2013–2016)
L. Matano	40			

Calculation was done for the differences between the same seasons (see text).
