# Corrigendum: Will hypolimnetic waters become anoxic in all deep tropical lakes?

**DOI:** 10.1038/srep46974

**Published:** 2018-05-17

**Authors:** Takehiko Fukushima, Bunkei Matsushita, Luki Subehi, Fajar Setiawan, Hendro Wibowo

Scientific Reports 7: Article number: 4532010.1038/srep45320; published online: 05 28 2017; updated: 05 17 2018

This Article contains errors in Reference 25 and 26, which are incorrectly given as follows:

‘Jacobson, P. *First Toba, now Maninjau: another mass fish death hits an Indonesian lake. Mongabay*. https://news.mongabay.com/2016/09/first-toba-now-maninjau-another-mass-fish-death-hits-an-indonesian-lake/cited on Sep. 28, 2016 (2016).’

‘Jacobson, P. *Why did millions of fish turn up to dead in Indonesia’s giant Lake Toba. Mongabay*. https://news.mongabay.com/2016/08/why-did-millions-of-fish-turn-up-dead-in-indonesias-giant-lake-toba/ cited on Sep. 28, 2016 (2016).’

The correct references are listed below as refs 1 and 2.
