# Supplementary material for: Will hypolimnetic waters become anoxic in all deep tropical lakes?
Source: Sci Rep. 2017 Mar 28;7:45320. doi: 10.1038/srep45320 (PMC5368600; doi:10.1038/srep45320)
Supplement: Supplementary Information [file srep45320-s1.pdf]

Will hypolimnetic waters become anoxic in all deep tropical lakes?

Takehiko Fukushima, Bunkei Matsushita, Luki Subehi, Fajar Setiawan, & Hendro Wibowo

Supplementary Fig. 1 Changes in vertical profiles of dissolved oxygen saturation. (1) Lake Toba North Basin, (2) Lake Toba South Basin, (3) Lake Maninjau, (4) Lake Singkarak, (5) Cirata Reservoir, (6) Lake Buyan, (7) Lake Batur, (8) Lake Matano

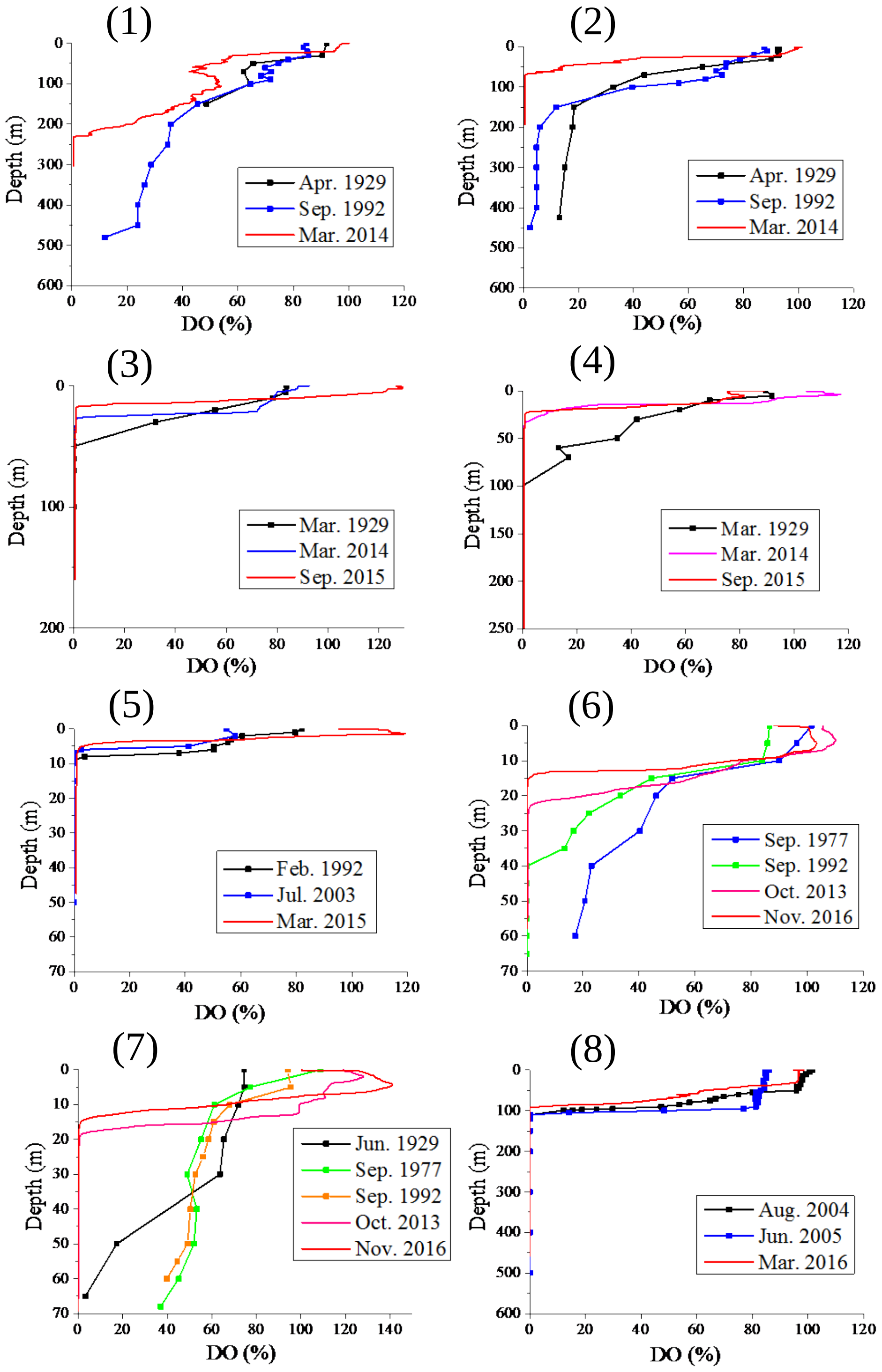

Supplementary Fig. 2 Changes in vertical profiles of water temperature. (1) Lake Toba North Basin, (2) Lake Toba South Basin, (3) Lake Maninjau, (4) Lake Singkarak, (5) Cirata Reservoir, (6) Lake Buyan, (7) Lake Batur, (8) Lake Matano

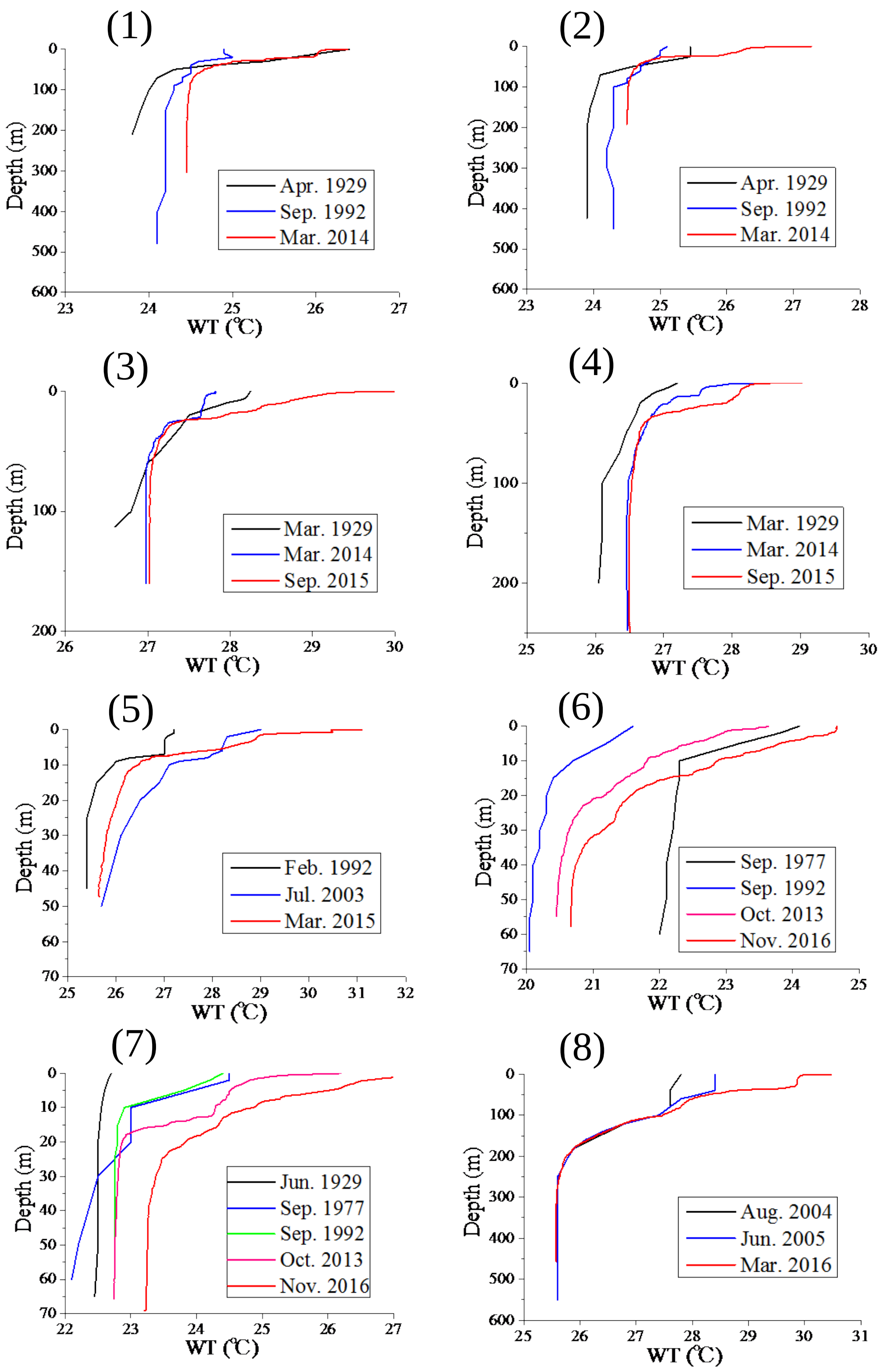

Supplementary Fig. 3 Vertical profiles of water density derivatives over depth in the eight water bodies by our survey

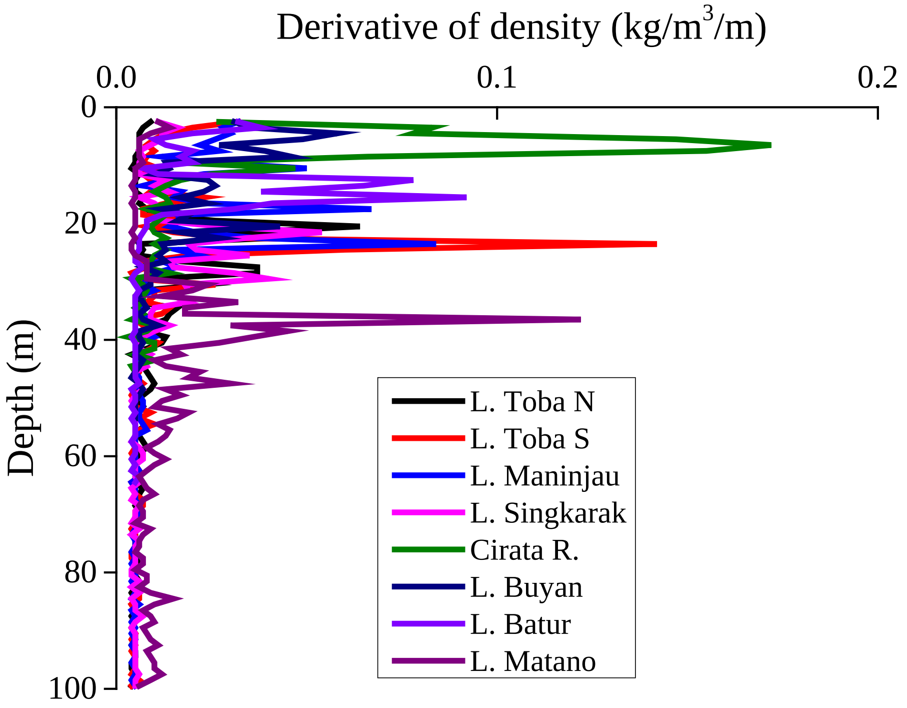

Supplementary Fig. 4 Vertical profiles of corrected electric conductivity adjusted to 25 °C (EC25) in the eight water bodies by our survey

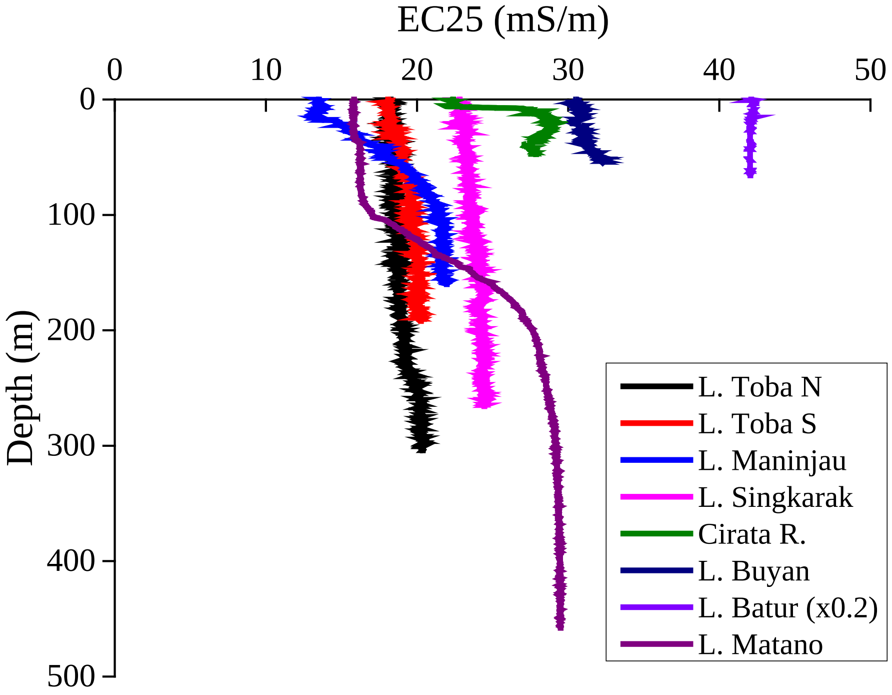

Supplementary Table 1 Data sources of water quality. Periods A, B and C indicate the results obtained in 1929, 1975-2005 and 2013-2016, respectively. Numbers indicate the order of measurements in the respective periods.

Supplementary Table 1 Data sourses of water quality

Lake name	A (1929)	B (1975-2005)			C (2013-2016) (This study)	
	No. 1	No. 1	No. 2	No. 3	No. 1	No. 2
L. Toba North	Apr. 15, 1929[13]	Sep. 20, 1992[15]			Mar. 19, 2014	
L. Toba South	Apr. 1, 1929[13]	Sep. 19, 1992[15]			Mar. 19, 2014	
L. Maninjau	Mar. 11, 1929[13]				Mar. 16, 2014	Sep. 7, 2015
L. Singkarak	Mar. 7, 1929[13]				Mar. 17, 2014	Sep. 8, 2015
Cirata R.		Feb. 29, 1992[15]	Jul. 29-30, 2003[16]		Mar. 16, 2015	
L. Buyan		Mar. 2, 1975[14]	Sep. 30, 1975[14]	Sep. 3, 1992[15]	Oct. 20, 2013	Nov. 7, 2016
L. Batur	Jun. 21, 1929[13]	Feb. 19, 1975[14]	Sep. 28, 1977[14]	Sep. 1, 1992[15]	Oct. 23, 2013	Nov. 8, 2016
L. Matano		Aug.-Sep., 2004[17]	Jun.-Jul., 2005[17]		Mar. 26, 2016	

Supplementary Table 2 Changes in Secchi depth transparency (m)

Supplementary Table 2 Changes in Secche depth transparency (m)

Lake name	A (1929)	B (1992 or 1993)	C (2013 - 2016)
L. Toba North		15.0 (1992)	6.5 (2014)
L. Toba South	11.5 (1929)	13.5 (1992)	6.4 (2014)
L. Maninjau	6.5 (1929)	3.4 (1992)	2.1 (2014)
L. Singkarak	5.5 (1929)	2.6 (1992)	4.4 (2014)
Cirata R.		1.2 (1992)	1.5 (2015)
L. Buyan		2.7 (1992)	2.6 (2013)
L. Batur		3.1 (1992)	1.3 (2013)
L. Matano		15.5 (1993)	16.8 (2016)

Supplementary information on dissolved oxygen conditions in other lakes

Our surveys during 2013-2016 included Lakes Tondano, Limboto, Towuti and Berantan and Jatiluhur Reservoir of which the vertical distributions of dissolved oxygen have not been previously reported. The observed shallowest depths of anoxic layers were shown below.

Lake Tondano (Sulawesi island) 18.7 m on Mar. 18, 2013
Lake Limboto (Sulawesi island) no anoxic layer on Mar. 20, 2013
Lake Towuti (Sulawesi island) 133 m on Mar. 27, 2016
Lake Berantan (Bali island) 16.6 m on Nov. 7, 2016
Jatiluhur Reservoir (Java island) 18.0 m on Jul. 15, 2014
